# The Multilevel Structure of Sulfonated Syndiotactic-Polystyrene Model Polyelectrolyte Membranes Resolved by Extended Q-Range Contrast Variation SANS

**DOI:** 10.3390/membranes9110136

**Published:** 2019-10-24

**Authors:** Maria-Maddalena Schiavone, Hiroki Iwase, Shin-ichi Takata, Aurel Radulescu

**Affiliations:** 1 Jülich Centre for Neutron Science (JCNS) at Heinz Maier-Leibnitz Zentrum (MLZ), Forschungszentrum Jülich GmbH, 85747 Garching, Germany; schiavonemariamaddalena@yahoo.it; 2 Neutron Science and Technology Center, Comprehensive Research Organization for Science and Society (CROSS), 162-1 Shirakata, Tokai, Ibaraki 319-1106, Japan; h_iwase@cross.or.jp; 3 Materials and Life Science Division, Japan Proton Accelerator Research Complex (JPARC), Tokai, Ibaraki 319-1195, Japan; shinichi.takata@j-parc.jp

**Keywords:** proton exchange membranes, semi-crystalline polymers, small-angle neutron scattering

## Abstract

Membranes based on sulfonated synditoactic polystyrene (s-sPS) were thoroughly characterized by contrast variation small-angle neutron scattering (SANS) over a wide Q-range in dry and hydrated states. Following special sulfonation and treatment procedures, s-sPS is an attractive material for fuel cells and energy storage applications. The film samples were prepared by solid-state sulfonation, resulting in uniform sulfonation of only the amorphous phase while preserving the crystallinity of the membrane. Fullerenes, which improve the resistance to oxidation decomposition, were incorporated in the membranes. The fullerenes seem to be chiefly located in the amorphous regions of the samples, and do not influence the formation and evolution of the morphologies in the polymer films, as no significant differences were observed in the SANS patterns compared to the fullerenes-free s-sPS membranes, which were investigated in a previous study. The use of uniaxially deformed film samples, and neutron contrast variation allowed for the identification and characterization of different structural levels with sizes between nm and μm, which form and evolve in both the dry and hydrated states. The scattering length density of the crystalline regions was varied using the guest exchange procedure between different toluene isotopologues incorporated into the sPS lattice, while the variation of the scattering properties of the hydrated amorphous regions was achieved using different H_2_O/D_2_O mixtures. Due to the deformation of the films, the scattering characteristics of different structures can be distinguished on specific detection sectors and at different detection distances after the sample, depending on their size and orientation.

## 1. Introduction

Owing to their high conversion efficiency, high power density, low weight and volume, fast startup time, low operating temperature (below 100 °C), and clean exhaust, polymer electrolyte membrane (PEM) fuel cells (PEMFC) are considered an attractive energy conversion technology for transportation applications, as demonstrated by the prototyped fuel cell vehicles and announced near future production plans by almost all major car manufacturers [1,2]. With the potential to become an alternative to the currently used fossil fuel technologies in light-duty transportation, and thus reduce the dependence on conventional fuels and the CO_2_ emissions, PEMFCs show not only economical, but also environmental benefits.

In a simplistic picture of the working principle of a PEMFC, the PEM separates the anode from the cathode and conducts at very high rates protons, which emerge from hydrogen oxidation reaction that is facilitated by the catalyst in the anode chamber [3]. However, the transport phenomenon in the PEM is a complex process because, on one hand, besides protons being the only ones to be transferred through the membrane, other species are produced at the anode too, and, on the other hand, the leaking of fuel (hydrogen) and oxidant (air) from the anode chamber to the counterpart must be prevented [4]. Moreover, a very efficient PEMFC requires the membrane to function in a hydrated state, which usually lowers the mechanical strength. Therefore, great efforts have been spent in the last years to develop and characterize materials that are approaching the properties of an ideal “separator” [5], and to understand and optimize the water management in different components of the PEMFC [6,7,8]. PEM materials should be characterized by a nanoscale phase separation into hydrophilic domains and hydrophobic regions, which is a combination that enables a high conductivity and provides a good chemical and mechanical stability, and thus membrane durability. Perfluorosulfonated ionomers (PFSI) present high performances and stability in PEMFC operational conditions. Among them, the Nafion (Du Pont^TM^) is the most well-known material, and was established as benchmark for such applications [9]. However, despite their excellent properties, the PSFI materials present several drawbacks such as their high cost, lack of safety, and the requirement of supporting equipment during manufacturing and use [10]. Furthermore, they have limitations under operating conditions at high temperature (>80 °C) and low relative humidity (RH), when a decrease in conductivity appears due to dehydration of the membrane at the anode side [11]. Moreover, free radicals such as hydroxyl and hydroperoxyl are produced during the operation of the PEMFC as a result of the reaction of hydrogen and oxygen on the electrodes or the decomposition of hydrogen peroxide with metal contaminants in the membrane. These radicals initiate processes of chemical degradation that affect the durability and the lifetime of the PEM [12,13]. Thus, the PSFI membranes seem not to be the ideal choice for the commercialization of PEMFC technology. Several approaches have been proposed to overcome these issues, considering both the improvement of the properties of the Nafion membranes and the development of alternative membrane materials with similar or better performance. However, there is always a trade-off between improving one or another of these properties [12]. The ion-exchange capacity (IEC), and hence the conductivity, can be increased by adding ionic groups to the Nafion, but this may lead to deterioration of the mechanical properties of the membrane due to excessive swelling. In addition, the incorporation of hygroscopic inorganic fillers into Nafion improves the mechanical and thermal stability and the retention of water within the membrane, but this leads to a decrease in the number of sulfonate groups per unit volume, which reduces the conductivity. Recently, it has been demonstrated that the incorporation of CeO_2_ and amine-functionalized carbon nanotubes (ACNTs) into the Nafion matrix has a bifunctional consequence toward improving the proton transport due to acid–base interaction between the proton donor sulfonic group and proton acceptor amine group without the aid of water and the mitigating chemical degradation of membranes due to free radical reduction, which is promoted by the ceria [12].

Alternative low-cost materials that present similar conductive and chemo-mechanical properties as the PFSI membranes are continuously searched for. Different crystalline-amorphous polymer architectures and the interrelation of their properties with the microphase separation structures, such as crystalline domains, the formation of conducting regions, and the distribution of ionic groups and water in the conducting regions were extensively studied in recent years [13,14,15,16,17,18,19,20]. 

Given the recent developments, which enable a controlled sulfonation of only the amorphous phase, preserving thus the crystallinity of the material [21], and an improved resistance to oxidation decomposition when fullerenes are added [22], the sulfonated syndiotactic polystyrene (s-sPS) in its β-form is a good potential candidate for some PEMFC applications, as it presents a high proton conductivity comparable to Nafion [23], high chemical and thermo-mechanical stability, and a low cost [24]. The preparation of a s-sPS membrane should start from the δ-form (clathrate with guest molecules) to enable a homogeneous sulfonation of only the amorphous regions, and can be subsequently transformed into the thermodynamically stable β-form by chemical/annealing treatment [23,25]. sPS-based membranes may also find application in the energy storage field, for increasing the safety of high temperature operating Li-ion batteries, for example [26].

The nanomorphology of PEM materials and the elucidation of water domains and conductive paths at the microscopic level are highly debated topics. Even in the case of Nafion, several microstructural models are still under consideration [27]. In a previous work [28], we reported a detailed microstructural characterization of highly sulfonated membranes (above 50% sulfonation degree) based on an s-sPS δ-clathrate co-crystalline form [29,30]). The microstructural characterization was carried out by small-angle neutron scattering (SANS) during the complex preparation procedure of the membranes, from the production of the sPS film samples in α-form followed by clathration with toluene guest molecules (to yield the δ-form [31]), sulfonation and in situ hydration under controlled RH by using a humidity chamber (Anton Paar). The use of uniaxially deformed sPS films enabled the assignment of the scattering signals observed on different sectors of the SANS detector to specific morphologies that formed and evolved in the sample during the clathration, sulfonation, and hydration/dehydration processes. Besides the structural characterization of the crystalline lamellar stacks and the water cluster morphologies evolving in the amorphous regions with the increasing RH, information on the mechanical strength and stability of the membranes due to the preservation of crystallinity could be assumed from the interpretation of the SANS data and confirmed at a later time in subsequent experiments by wide-angle X-ray diffraction (WAXD).

In this paper, we report a structural investigation by contrast variation SANS on uniaxially deformed s-sPS films containing the crystalline δ-form that were prepared with different degrees of sulfonation. The films were loaded with fullerenes, in order to reproduce the conditions proposed for such materials to reach the optimal chemical stability in a PEMFC environment. Although still rarely mentioned in the fuel cell applications, PEMs based on fullerene-hydrocarbon composites are under development have shown at the laboratory scale an improved stability due to the radical scavenging role that is played by fullerenes and the interfacial hydrogen bonding between the functionalized fullerenes and the host matrix [22,32]. A first insight on the microstructure of s-sPS membranes incorporating fullerenes can be obtained from the combined analysis of the data obtained by SANS and other complementary methods, such as UV-Vis spectroscopy and wide-angle X-Ray diffraction (WAXD).

**Figure 1:**
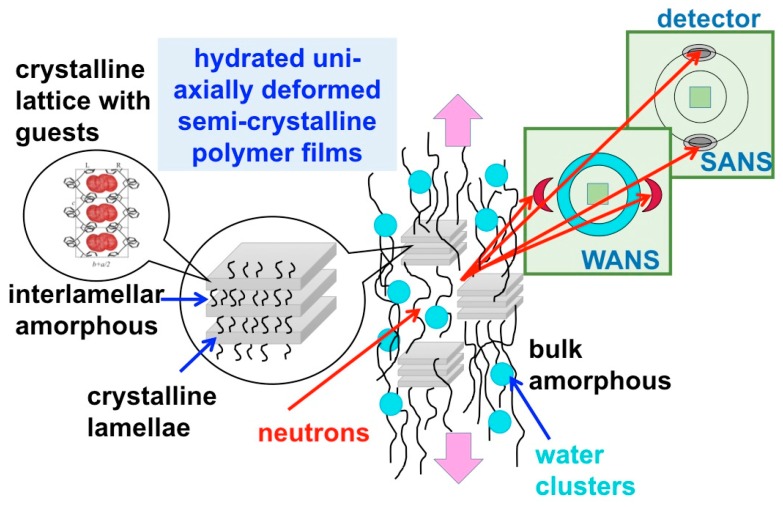
Schematic view of the experimental approach used in this study: the uniaxially polymer film deformation is indicated by the pink arrows, while the morphologies occurring at different length scales, which are shown in the left side of the scheme, yield on the two-dimensional small-angle neutron scattering (SANS) detector scattering features that appear at different scattering angles, either in the wide-angle (WANS) or small-angle (SANS) regime, as isotropic or localized details.

The SANS experiments were carried out over a wide wave-vector transfer Q-range, between 0.001 and 2 Å^−1^, which enabled the observation of scattering features from morphologies and structures formed at very different length scales in the membranes, such as the 010 crystalline peak of the crystalline lattice characteristic of the lamellae in the crystalline domains, the ionomeric peak representing the structural correlation length for the ionic hydrophilic domains in the amorphous regions, the interlamellar peak representing the correlation length between the crystalline lamellae, the form factor of the water domains, and the large-scale fractal of the membranes. Thus, a very extended length scale, from a few Å to nm, could be explored in this investigation. Due to the uniaxial deformation of the films, some of the scattering details from these morphologies appear distributed on specific detector sectors, such as the features that are characteristic of crystalline domains: the 010 crystalline peaks appear on equatorial sectors, while the interlamellar peaks appear in the meridian sectors direction. Other morphologies yield scattering that is isotropically distributed over whole detection area, such as the features from the amorphous regions, thus the ionomer peak and the water domains form factor. A schematic view of the experimental geometry approach used in this study, and of the novelty we have implemented compared to our previous approach [28], is shown in Figure 1.

In order to minimize the SANS incoherent background, deuterated sPS films have been used in the study. The contrast variation method was involved to vary the scattering length density (SLD) of different film components in a controlled way, i.e., by using either deuterated or protonated species for the guest molecules in the crystalline regions, or different H_2_O/D_2_O mixtures for the hydration of the membranes. Thus, the formation and evolution of all these morphologies during the hydration process could be fully understood following the model interpretation of the scattering curves, which were averaged over the meridian and equatorial sectors for each contrast condition.

## 2. Materials and Methods 

### 2.1. Materials

The preparation and subsequent treatment-clathration, sulfonation, guest exchange in the crystalline region—of uniaxially deformed deuterated syndiotactic polystyrene films were done following a procedure that is extensively described in [28]. Films with variable degrees of sulfonation were produced via a so-called solid-state sulfonation procedure that allowed a uniform sulfonation of the phenyl rings of the amorphous phase and preserved the crystalline δ-form [21]. The thickness of the films was about 100 μm. To vary the neutron scattering contrast, either deuterated or protonated toluene were loaded as the guest in the clathrate form, either before or after sulfonation, by dipping the films for 1 d in solvent, followed by drying at 40 °C under vacuum for a couple of hours. Fullerenes C60 or C70 were uploaded in some of the s-sPS membranes by dipping the samples for more than three weeks in saturated solution of fullerenes and protonated toluene. During the SANS experiments, two films of different composition were subjected to in situ controlled hydration under vapors of different H_2_O/D_2_O mixtures, by using a humidity chamber [28]. All reagents were purchased from Sigma-Aldrich (Munich, Germany) and used as received. The D_2_O was obtained from Cambridge Isotope Laboratories (purity D 99.8%).

### 2.2. Methods

The degree of sulfonation was checked at the neutron prompt-gamma activation analysis (PGAA) instrument of Technical University München (TUM) installed at the Heinz Maier-Leibnitz Zentrum (MLZ), Garching, Germany. Full descriptions of the experimental method and data interpretation can be found in [28].

Qualitative analysis of the sulfonation, the incorporation of fullerenes, and the degree of crystallinity were checked by Fourier-transform infrared (FTIR) spectroscopy using a PerkinElmer (Spectrum Two, Rodgau-Jügesheim, Germany) spectrometer equipped with a triglycine sulfate (TGS) detector. The scanned wavenumber range was 4000–400 cm^−1^.

WAXD analysis of sulfonated films with and without the addition of fullerenes was done prior to the SANS experiments in the range of 2θ between 5 and 35° by means of an X-ray powder diffractometer Bruker 2^nd^ Gen-D2 Phaser (Cu-source) of Jülich Centre for Neutron Science (JCNS) at MLZ (Karlsruhe, Germany). The degree of crystallinity was determined as 100 A_c_/(A_c_ + A_a_), where A_c_ and A_a_ are the areas determined by resolving the diffraction pattern according to [33], and can be considered proportional to the crystalline and amorphous fractions of the polymer. 

UV-Vis analysis of the same films was carried out with a Cary 100 SCAN UV-Vis Varian spectrometer of JCNS (Palo Alto, CA, USA) at MLZ with the films placed in a specific holder with quartz windows. The spectra were collected in the range 200–800 nm at a resolution of 100 nm/min. 

Preliminary SANS measurements were carried out at the KWS-2 high intensity/extended-Q range pinhole SANS diffractometer (Forschungszentrum Jülich GmbH, Jülich, Germnay) of JCNS at MLZ [34]. A Q-range between 0.02 and 0.7 Å^−1^ was covered by using two sample-to-detector distances, L_D_ = 1.5 and 4 m and a neutron wavelength λ = 4.5 Å. The film samples were placed in the beam by means of sandwich-type cells with quartz windows.

Extended Q-range SANS experiments have been performed at the time-of-flight (TOF) SANS diffractometer TAIKAN, at the Material and Life Science Experimental Facility (MLF) of the Japan Proton Accelerator Research Facility (J-PARC), Tokai, Japan [35]. A Q-range between 0.008 and 2 Å^−1^ was covered by using a broad neutron wavelength range λ = 0.7 to 7.8 Å, and the simultaneous use of only the small-angle and middle-angle detector banks (due to restrictions imposed by the sample environment). Involving the additional use of the wide-angle and back-scattering detector banks, which are available at this instrument, too, a Q_max_ = 20 Å^−1^ could be otherwise reached in a single measurement for a sample geometry that would allow the detection of the scattered neutrons in a wide angular range. At the small-angle scattering diffractometer TAIKAN, the film samples were exposed to the in situ controlled hydration within the range RH = 50 to 80% by means of an Anton-Paar humidity chamber [28]. Contrast variation SANS measurements were carried out by exposing the sample to mixed H_2_O/D_2_O vapors for different ratios of the two components at RH = 80%.

For both SANS instruments, the raw data was treated by a standard corrections and reduction procedure [34,35], and then calibrated in absolute units by using a Plexiglas (at KWS-2) or a glassy-carbon (at TAIKAN) secondary standard. The corrected and calibrated 2D data were integrated into one-dimensional intensity over equatorial or meridian sectors of a 20° width. The SLD values for different compounds in the s-sPS film morphology are listed in Table 1, as it was calculated or taken from literature.

### 2.3. Data Analysis 

Supposing we have N identical particles of volume V_p_, which are located at random positions and random orientations in the sample, then NV_p_ = ϕV_sample_, where ϕ is the volume fraction of the scattering particles in the sample. The contribution to the small-angle scattering intensity from these particles that are decorated with a constant contrast factor Δρ is:

(1) $$I\left(Q\right)=\phi {\;\Delta \rho }^{2}\;V_{p}\;P\left(Q\right)\;S\left(Q\right)+\mathrm{Bckgd}$$

where P(Q) represents the particle form factor, which relates to the intraparticle correlations, and S(Q) represents the structure factor, which denotes the interparticle correlation effects. The contrast Δρ = ρ_p_ − ρ_env_ is the difference between the SLD of the scattering particles ρ_p_ and their environment ρ_env_, where the environment can be a solvent, a film, or a metallic matrix. Usually, the factor (ϕ Δρ^2^ V_p_) is called the “forward scattering” I_0_ from the ensemble of scattering particles. The term Bckgd represents a constant background, which arises mostly from the incoherent scattering contribution, and can be observed as a constant level at high Q.

In the current study, we used the combination of the form factor and the structure factor to describe the scattering from the water domains in the amorphous phase of the s-sPS films and the lamellar stacks in the crystalline regions. For the water clusters, the spherical form factor:

(2) $$P_{\mathrm{sph}}\left(Q\right)={\left[3\frac{\sin\left(\mathrm{QR}\right)-\mathrm{QR}\;\cos\left(\mathrm{QR}\right)}{{\left(\mathrm{QR}\right)}^{2}}\right]}^{2}$$

was combined with the hard-sphere structure factor [37]:

(3) $$S\left(Q,{\;R}_{\mathrm{HS}}\right)={\left[1+24{\;\eta }_{\mathrm{HS}}\;G\left(R_{\mathrm{HS}}Q\right)/\left(R_{\mathrm{HS}}Q\right)\right]}^{-1}$$

where R is the radius of the spherical cluster, R_HS_ is the “hard sphere” radius of the interaction potential, and η_HS_ is the volume fraction of hard spheres. The function G(R_HS_Q) has a complicated analytical dependence on η_HS_ [37]. The lamellar stacks consisting of oriented crystalline lamellae that alternate with amorphous interlamellar regions was described by the two-dimensional crystalline-amorphous form factor [38], where crystalline lamellae have amorphous layers attached on both faces:

(4) $$P_{\mathrm{lam}}\left(Q\right)={\left({\Delta \rho }_{\mathrm{cr}}P_{\mathrm{cr}}\left(Q\right)+{\Delta \rho }_{\mathrm{am}}P_{\mathrm{am}}\left(Q\right)\right)}^{2}\frac{D\left({\mathrm{QR}}_{l}/2\right)}{\left({\mathrm{QR}}_{l}/2\right)}{\left({\pi R}_{l}^{2}\right)}^{2}$$

in combination with the paracrystalline structure factor:

(5) $$S_{\mathrm{para}}\left(Q\right)=\frac{\sin h\left(Q^{2}\sigma_{D}^{2}/4\right)}{\cosh\left(Q^{2}\sigma_{D}^{2}/4\right)-\cos\left({\mathrm{QL}}_{D}\right)}$$

where L_D_ is the interlamellar distance (periodicity), σ_D_ is its dispersion, and R_l_ is the lateral size of the lamellae. Since we are dealing here with a ternary system consisting of the crystalline lamellae, the interlamellar amorphous region, and the surrounding bulk amorphous region, the contrast factor from Equation (1) was included in the form factor definition in Equation (4), with the aim to express the difference in SLD between these three components. The Dawson function D(u) exhibits the following asymptotic behavior: for u→∞, 2D(u)→1/u^2^ and for u→0, D(u)/u→1. The partial form factor of the crystalline lamellae P_cr_(Q) is given by:

(6) $$P_{\mathrm{cr}}\left(Q\right)={\left[\frac{\sin\left(\frac{\mathrm{Qd}}{2}\right)}{\left(\frac{\mathrm{Qd}}{2}\right)}\right]}^{2}$$

with d represents the lamellar thickness, while the partial form factor of the interlamellar amorphous layers P_am_(Q) has a complicated analytical definition [38] that depends on both the lamellar thickness d and the thickness of the interlamellar layer L_b_. Hence, L_D_ = d + L_b_. The SLD for three distinct regions of the modeled morphology were explicitly considered in the fitting procedure: ρ_lam_ is the SLD of the crystalline sPS, ρ_inter-lam_ is the SLD of the sulfonated interlamellar amorphous region, and ρ_bulk_ is the SLD of the sulfonated bulk amorphous region. Thus, the contrast factors in Equation (4) are Δρ_cr_ = ρ_lam_ − ρ_bulk_ and Δρ_am_ = ρ_inter-lam_ − ρ_bulk_. These three regions are affected in a different way by hydration. The bulk and interlamellar amorphous regions are hydrated, so they swell upon water uploading, as reported before [28]. Therefore, their SLD is changed according to:

(7) $$\rho_{\mathrm{bulk},\;\text{inter-lam}}=\left(\phi_{\mathrm{pol}}\rho_{\text{s-sPS}}+\phi_{\mathrm{water}}\rho_{\mathrm{water}}\right)$$

with ρ_s-sPS_ and ρ_water_ representing the SLD for the sulfonated polymer and the H_2_O, D_2_O, or H_2_O/D_2_O mixtures, as defined in Table 1, and ϕ_pol_ and ϕ_water_ representing the volume fractions of polymer and hydrated water in the swollen amorphous regions. Disregarding the free volume in the amorphous polymer, which typically is very small [39], we can roughly assume that ϕ_water_ = 1 − ϕ_pol_ in these regions.

The global water fraction in the amorphous phase can be determined from the interpretation of the “forward scattering” from the water domains. However, there is an unknown partition of water between the bulk and interlamellar amorphous regions. Moreover, due to the loading with guest molecules, either protonated toluene or fullerenes, the SLD of the lamellar region is lower than that of the crystalline sPS (Table 1). Therefore, in the fitting procedure of the experimental data from the lamellar stacks (Equations (1), (4)–(6)), the SLD parameters ρ_lam_ and ρ_inter-lam_ were considered free parameters. The ρ_bulk_ in this procedure was considered that of the amorphous sPS (Table 1). This is a reasonable assumption if we consider that the sulfonated segments of the sPS chains in the bulk region are contained in the water domains, thus not affecting the SLD of the amorphous segments in the vicinity of the lamellar stacks. The obtained value for ρ_inter-lam_ was further used for rationalization on the polymer volume fraction and water content in the interlamellar amorphous regions (Equation (7)). On the other hand, the crystalline lamellae are not hydrated; therefore, the ρ_lam_ delivered by the fitting procedure was used for the estimation of the amount of guest molecule included in the crystalline region, in a similar way as shown in Equation (7), with the guest molecule instead of water. According to [30], the amount of guest molecules included in the crystalline region may vary up to about 9% in case of the δ-clathrate form. Therefore, the SLD of the crystalline region in our films may decrease from the value reported in Table 1 for the crystalline sPS up to ρ_lam_ = 5.9 × 10^10^ cm^−2^, taking into account the contribution of the protonated toluene to the overall SLD of the crystalline region. 

## 3. Results and Discussion

### 3.1. Composition and Crystallinity Characterization.

From the quantitative point of view, PGAA delivered the sulfur-to-carbon (S/C) ratio of 0.065 and 0.155 for the two uniaxially deformed sPS films studied in this work. Following the method reported in [40], which was used also in our previous study [28], sulfonation degrees of S = 19.5 and 46.3% (molar fraction of sulfonated monomer units) were determined for the samples, which were later subjects of doping with C60 and C70 fullerenes, respectively. 

**Figure 2:**
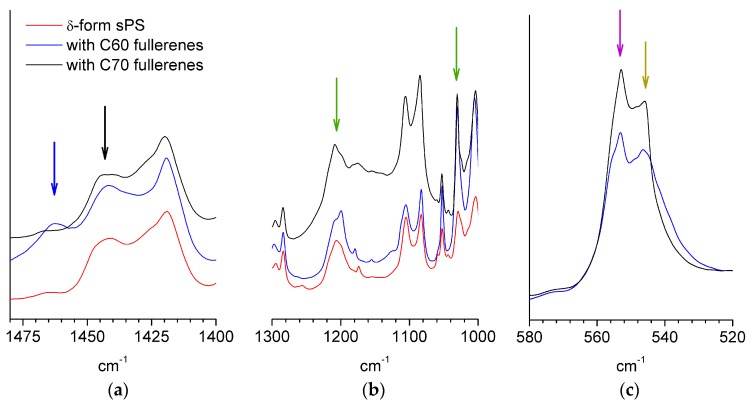
FTIR spectra from different synditoactic polystyrene (sPS) films: δ-form (red line), sulfonated and loaded with C60 fullerenes (blue line); sulfonated and loaded with C70 fullerenes (black line). The region where additional infrared ands to those from the polymer film are observed (blue and black arrows) is shown in panel (**a**), while the IR bands from the sulfonic ionic group (green arrows) are shown in panel (**b**). Panel (**c**) shows the bands characteristic of sPS: the arrows indicate the peaks that were considered to correspond to chain conformations in crystalline (purple) and amorphous (dark yellow) phases.

The FTIR spectra of the films doped with C60 (blue curves) or C70 (black curves) fullerenes are shown in Figure 2 in parallel to that from a δ-form sPS film (red curve). Due to the multitude of characteristic bands of sPS, only weak differences between fullerene-free and fullerene-doped films could be observed, similar to those indicated in the region 1440–1470 cm^−1^ (Figure 2a). However, it is not clear that the origin of these infrared bands can be attributed to fullerenes. An experimental FTIR characterization of fullerenes in bulk or functionalized polymers can be found in [41] for C60 or [42] for C70, while theoretical calculations were done in [43]. 

The region of symmetric and asymmetric stretching of the SO_3_^−^ group is shown in Figure 2b. The bands corresponding to the sulfonic group were observed at around 1240 and 1040 cm^−1^, which is in good agreement with early reports on sulfonated copolymers [44,45]. 

From the evaluation of the bands that are characteristic to crystalline and amorphous sPS, the amount of polymer in the ordered TTGG sequences can be satisfactorily estimated following the procedure described in [46]. This would give a rough indication of the crystallinity of the sample. Information about the FTIR spectra from deuterated sPS either in film or solution/gel samples is very scarce in the literature. Therefore, for this exercise, we considered the bands in the range 500–600 cm^−1^, which is a range that was never discussed before in the case of the deuterated sPS system and, which, according to our investigations on crystallization from solution (results to be published soon), contains information about either the helical or amorphous polymer chain conformation. Assuming that the two peaks shown in Figure 2c for each sample could be assigned to the sPS in crystalline (556 cm^−1^) or amorphous (545 cm^−1^) phases, the fraction of conformationally ordered polymer obtained from the interpretation of the areas of the peaks would be about 35 and 22% for the films with a higher sulfonation degree (loaded with C70) and lower sulfonation degree (loaded with C60), respectively. A more accurate evaluation of the crystallinity can be obtained from the WAXD spectra.

**Figure 3:**
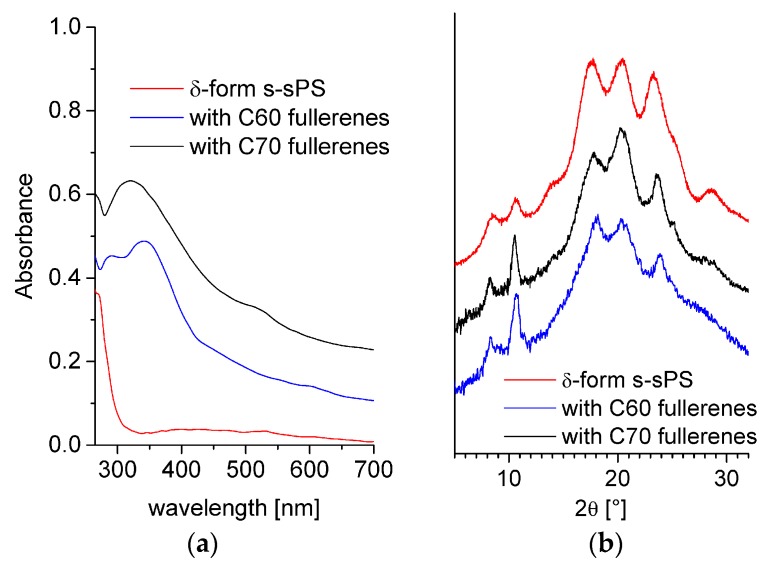
UV-Vis (**a**) and WAXD (**b**) spectra from different sPS films. The lines indicate sulfonated film with crystalline δ-form (red line), sulfonated (with crystalline δ-form) and loaded with C60 fullerenes (blue line), and sulfonated (with crystalline δ-form) and loaded with C70 fullerenes (black line).

In Figure 3a, the UV-Vis absorption spectra of the three samples are shown in parallel. The polymer characteristic absorption features occur below 300 nm; therefore, the strong but rather featureless absorption observed in the region 300–700 nm in the case of fullerenes-doped polymers may be considered indicative for the incorporation of fullerenes in the membranes. Although some broad features may be observed in the 300–350 nm and 500–600 nm regions, however, the characteristic absorption bands for C60 or C70 fullerenes [42,47,48] are not clearly visible in these spectra. At this stage of the study, we do not have yet a clear explanation for this observation, remaining that the behavior of fullerenes incorporated into the s-sPS by the dipping of polymer films in saturated fullerene solution will be investigated in detail in a forthcoming study. Nevertheless, the prompt-gamma activation analysis (PGAA), FTIR, and UV-Vis results confirmed the successful sulfonation and loading of the sPS membranes with either C60 or C70 fullerenes.

WAXD spectra from the s-sPS films containing fullerenes are presented in the Figure 3b in parallel with the pattern from the s-sPS film with only protonated toluene loaded in the crystalline regions (clathrates). The pair of peaks at around 8 and 10° in 2θ is indicative for the formation of the crystalline δ-form of the clathrates [29,49]. The presence of these peaks in the patterns collected on the samples loaded with fullerenes indicates that the sPS crystalline habit is preserved in these samples, too. Their slight enhancement when C60 or C70 fullerenes were added may relate to anchoring of the fullerenes to the sPS chains, as it was discussed in [49]. The positions of the diffraction peaks are the same in all WAXD patterns, which indicates that the addition of fullerenes does not change the parameters of the polymer crystalline lattice. Since the formation of the crystalline regions in the membranes is a consequence of the initial exposure of the film samples to the action of toluene from solution [28] and the fullerenes may replace the toluene molecules in the δ-form sPS co-crystals via the guest exchange mechanism, we assume that the subsequent incorporation of fullerenes does not significantly alter the crystalline degree of the membranes. This is different from the situation of the films that are produced by common gelation of the sPS and fullerenes [49], when the incorporation of the fullerenes into the sPS lattice was evidenced by the slight changes that were observed in the position of the diffraction peaks from the polymer film containing fullerenes compared to the fullerenes-free polymer film. From the fitting of the diffraction patterns, the A_c_ (peaks) and A_a_ (background) areas were estimated, and the crystallinity of the membranes could be evaluated according to [33]. This was about 33 and 24% for the films with a higher sulfonation degree (loaded with C70) and lower sulfonation degree (loaded with C60), respectively, which is quite close to the results obtained from the analysis of the FTIR spectra. 

As the detailed analysis of WAXD spectra and of fullerenes behavior in s-sPS films is beyond the goal of this work, we limit ourselves here to a qualitative conclusion. Based on the characterization methods applied prior to SANS on our samples, we can confirm the presence of the crystalline δ-form in all films and the loading of samples with fullerenes, and we may only suppose that in some of the cavities between the sPS helices in the crystalline region, the protonated toluene initial guest was replaced by the fullerenes.

### 3.2. SANS on Dry Films

The main objective of this work is the microstructural characterization of the s-sPS membranes under hydration and the understanding of the formation and evolution of morphologies at te nanoscale and mesoscale. In our previous study [28], we discussed the indirect observation on the preservation of crystallinity in such systems during the chemical treatment and hydration procedures. A direct observation of this effect together with a detailed microstructural characterization of the membranes over a wide length scale can be achieved by using the contrast variation SANS over a wide Q-range. This enables the collection of the scattering features from the crystalline ordering in the range of nanometers up to the micrometer size large-scale domains in one experiment. For this purpose, the novel approach that involves careful SANS measurements at high angle was checked first at the KWS-2 SANS instrument in combination with the contrast variation method on two δ-clathrate sPS films with toluene as the guest in the cavities between the polymer helices: one film was investigated as produced, while the other one was investigated after subsequent sulfonation and loading with C70 fullerenes. 

**Figure 4:**
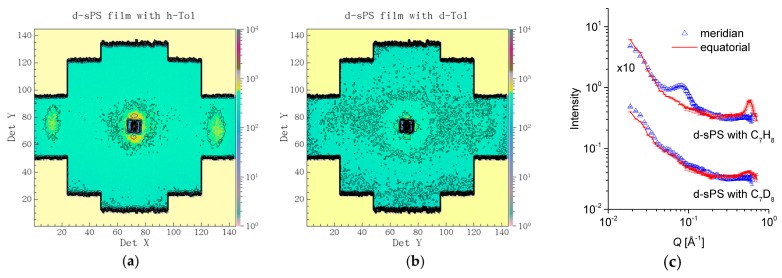
Two-dimensional SANS patterns from a deuterated sPS film clathrated with protonated toluene (**a**) or deuterated toluene (**b**), respectively, and the one-dimensional scattering profiles from the same samples averaged over the equatorial (line) or meridian (symbol) directions, respectively (**c**). The panels (**a**,**b**) show data collected at L_D_ = 2 m, while the experimental curves in panel (**c**) were obtained by merging data collected at L_D_ = 2 and 4 m.

In Figure 4, the scattering patterns from the uniaxially deformed sPS film containing clathrate co-crystalline δ-form with either protonated toluene or deuterated toluene are shown in two-dimensional (Figure 4a,b) and one-dimensional (Figure 4c) presentations, respectively, following the averaging over the meridian and equatorial sectors. In Figure 4a, two strong maxima can be observed in equatorial sectors at high angles, while two local maxima can be distinguished at low scattering angles in the meridian direction, above and below the beam stop, which is visible in the middle of the detector. These 010 reflections appear on the equator and the interlamellar reflections appear on the meridian, respectively, as depicted in the sketch presented in Figure 1. In Figure 4b, these features are either barely observable (the 010 reflections) or vanished (the interlamellar reflections). These features are also depicted by the one-dimensional patterns in Figure 4c. The 010 reflections are yielded by the correlation between polymer sheets that sandwich in between the guest molecules. The SLD of deuterated crystalline sPS is ρ = 6.47 × 10^10^ cm^−2^, while that of the toluene is ρ = 0.94 × 10^10^ cm^−2^ and ρ = 5.66 × 10^10^ cm^−2^ for the protonated and deuterated species, respectively. Thus, protonated toluene molecules hosted between deuterated sPS helices provides a high neutron contrast, which is most apparent from the correlation between 010 planes (see Figure 1, the crystalline lattice details). In case of using deuterated toluene guests, the neutron contrast is much lower, and the peaks on the equator become less obvious in the scattering pattern.

On the other hand, the interlamellar correlation peaks appear due to the difference in SLD between the amorphous and crystalline regions of the sPS co-crystals with guest molecules. The SLD of the amorphous sPS is ρ = 6 × 10^10^ cm^−2^. Loading the crystalline regions with protonated toluene will provide those regions with a lower SLD than the interlamellar amorphous regions, which will evidence the interlamellar correlations. In contrast, by using deuterated toluene, the difference in SLD between the crystalline lamellae and interlamellar amorphous regions will become much smaller than in the case of using protonated toluene. This will cause the interlamellar peaks on the meridian direction in the scattering patterns to vanish. Detailed SANS studies on the exchange of small guest molecules in sPS co-crystals are reported in [31,50]. 

**Figure 5:**
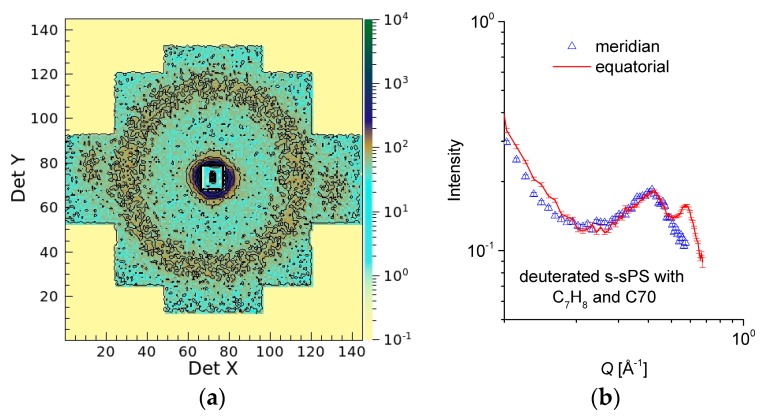
Two-dimensional SANS pattern from a dry s-sPS film containing the co-crystalline δ-phase with protonated toluene and loaded with C70 fullerenes (**a**) and one-dimensional scattering profiles from the same sample averaged over the equatorial (line) or meridian (symbol) directions, respectively (**b**). All data were collected at L_D_ = 2 m.

This contrast variation SANS investigation has proven that the status of the crystalline lattice can be monitored during the s-sPS sample treatment by observing the scattering features yielded at high angles. In Figure 5, we show the high Q scattering patterns from a dry s-sPS film. The film is characterized by a high sulfonation degree (S = 46.3%) and a crystallinity of roughly 35%, and was clathrated with protonated toluene and subsequently loaded with C70 fullerenes. The data are presented two-dimensionally (Figure 5a) and averaged over the equatorial and meridian sectors (Figure 5b). The 010 reflections are well visible in the equatorial sectors, while the ionomer peak, which for the dry membrane is indicative of the mean distance between the sulfonic ionic clusters [51], shows an isotropic distribution. The interlamellar reflections appear at much lower Q values in the case of the sulfonated samples, and are thus not visible in this experimental configuration. This is due to the swelling of the interlamellar amorphous regions, as already reported in [28]. A correlation distance of ξ_ion_ = 2π/Q_ion_ = 14.95 Å was obtained from the evaluation of the ionomer peak position in Q. This distance is smaller than the one determined for dry Nafion [52]. Taking into account the fact that the neutron SLD of fullerenes is very different from that of protonated toluene, but close to that of deuterated toluene [53], we may conclude that the replacement of the initial protonated toluene guest in the deuterated sPS crystalline region by the subsequently loaded fullerenes took place to a very small extent only, since the scattering features characteristic of the crystalline lattice were not affected apparently. Otherwise, the 010 reflections should have been reduced drastically, as in the case of using deuterated toluene as the guest in the sPS co-crystals. Thus, the scattering features from the crystalline regions in the s-sPS films can be still observed after the loading of samples with fullerenes.

### 3.3. SANS on Hydrated Films—Variation of Hydration Level

With this information at hand, two s-sPS samples with different degrees of sulfonation and crystallinity, which were loaded with either C60 or C70, were investigated at the TOF SANS diffractometer TAIKAN during hydration at different RH levels and with different mixtures of H_2_O/D_2_O. 

**Figure 6:**
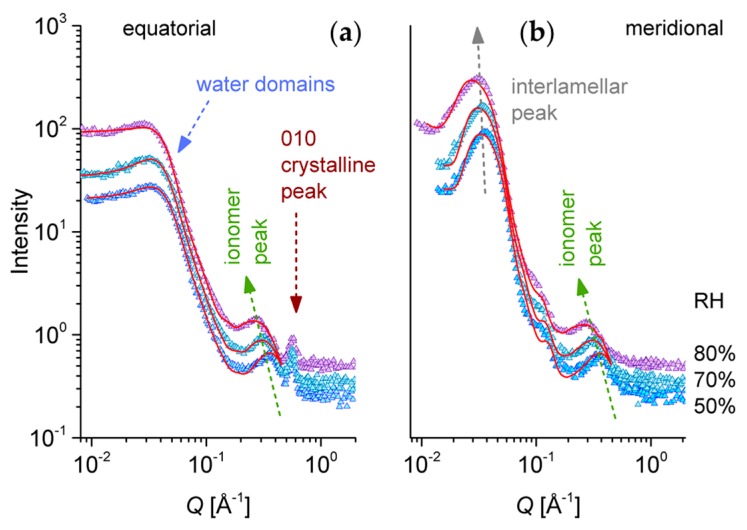
One-dimensional SANS patterns from the high sulfonated s-sPS film containing the co-crystalline δ-phase with protonated toluene and loaded with C70 fullerenes hydrated at different RH levels. Experimental data (symbols) averaged over the equatorial (**a**) or meridian (**b**) directions are shown separately, with the lines corresponding to the model interpretation of the scattering profiles, as discussed in text. The main structural features and their behavior with the variation of RH are indicated.

Figure 6 presents a selection of one-dimensional scattering data from the same s-sPS sample that was discussed in Figure 5, and which was hydrated with H_2_O at different RH levels. The data were averaged over the meridian and the equatorial sectors. The scattering patterns present three distinct peak-like features, which are observable for all hydration levels. These features are indicative of structural levels occurring at different length scales in the complex morphology of the polymer films. In the high Q range, the 010 crystalline peak appears in the equatorial sectors at around Q_010_ = 0.6 Å^−1^, as in the case of the sPS clathrates (Figure 4c) and the dry s-sPS sample (Figure 5b). This peak, which denotes a mean repeating distance between the sPS sheets of about 10–11 Å, does not change its position and intensity with the increase of the RH. This observation led to the conclusion that the hydration does not affect the crystalline structure. Again, the neutron contrast is provided by the protonated toluene guest molecules, which occupy the cavities between the deuterated s-PS helices to a larger extent.

**Figure 7:**
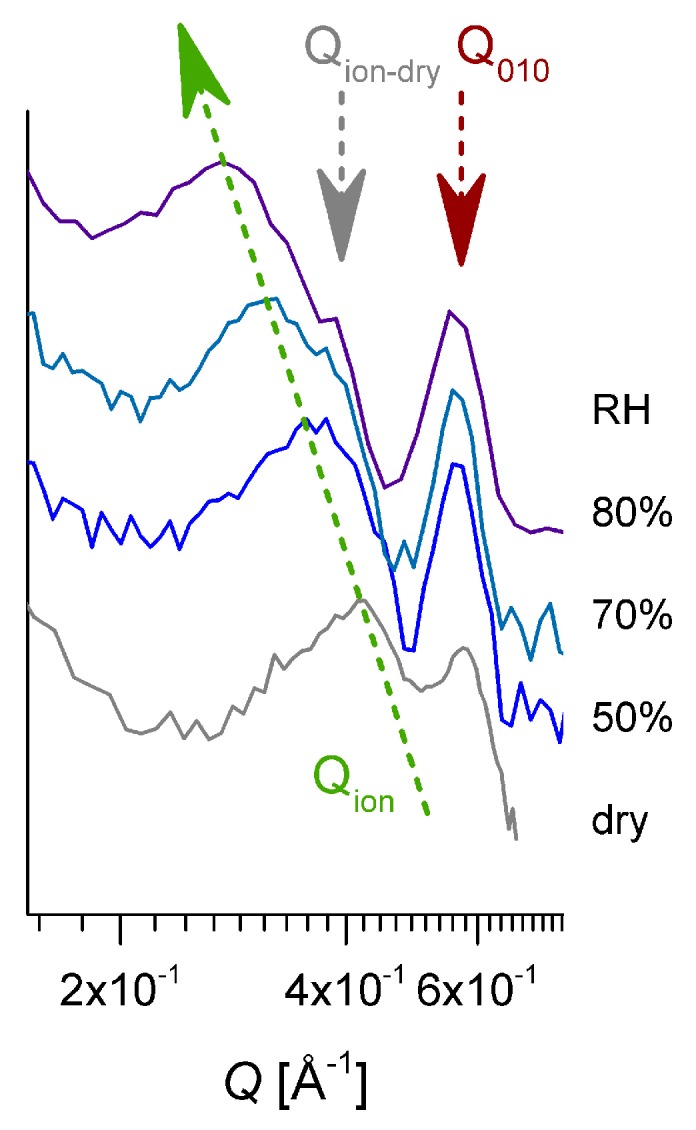
The high-Q range of the SANS patterns on the equatorial direction reported in Figure 6. The pattern from the membrane in a dry state was added. The green arrow indicates the variation in the position of the ionomer peak in increasing the hydration, and the dark red arrow marks the 010 reflection, while the gray arrow points to the ionomer peak position characteristic of the membrane in a dry state.

The ionomer peak is present in data on both the meridian and equatorial sectors, as it represents a scattering feature characteristic of the hydration occurring in amorphous regions, and is thus isotropically distributed on the detection area. The peak position Q_ion_ depends on the level of the film hydration [54,55]; thus, it moves toward lower values of Q with the increasing RH. A detailed presentation of the high Q scattering range from dry and hydrated films is given in Figure 7. In our sample, the correlation between the hydrated ionic clusters increases from about ξ_ion_ = 14.95 Å for dry film to about ξ_ion_ = 23.7 Å for hydrated film at RH = 80%. A close inspection of the ionomer peak profile reveals a shoulder-like feature on the high Q side of the peaks, which becomes clearer with increasing humidity, due to the shift of the peak position to lower Qs. The Q-position of this shoulder seems to remain constant, regardless of the RH, and corresponds to the Q-position of the ionomer peak that is characteristic of a dry membrane. Apparently, the part of the ionic clusters that gives rise to the occurrence of the ionomer peak in dry conditions is still not hydrated, even for higher RH values.

In the low Q region, the interlamellar peak characteristic of the oriented lamellar stacks (Figure 1) can be observed in the meridian scattering patterns. The peak position Q_lam_ moves only slightly to lower Q values with increasing RH, and denotes an interlamellar correlation of about 170–200 Å. In contrast, the equatorial scattering patterns exhibit at low Q a kind of plateau and a shoulder-like feature at around Q = 0.05 Å^−1^, which resemble characteristics of weakly correlated spherical morphologies. We propose that they represent loosely correlated large hydrated regions that include the ionic clusters. The scattering from these water domains should appear isotropically on the detector. However, in the meridian sectors, the scattering from the lamellar stacks is superimposing over it.

The scattering from these sulfonated s-sPS films is characterized by a high sulfonation degree and a relatively high crystallinity, which are loaded with C70 fullerenes, and resemble that from the fullerene free s-sPS films discussed in [28]. We may conclude that the partition of fullerenes between the amorphous and crystalline regions of these s-sPS films has a negligible effect on the scattering properties of the samples. As qualitatively concluded before, the C70 fullerenes seem to be located mostly in the amorphous regions rather than in the co-crystalline phase. 

On the other hand, the scattering from fullerenes or fullerene assemblies dissolved in solution or amorphous polymer environment, similar to the deuterated polystyrene in the present case, is very weak [56]. Therefore, we can consider it negligible compared to the contribution from other morphologies that form and evolve in our samples during the sulfonation and hydration processes. The experimental results in Figure 6 were interpreted via structural models: the data on equatorial sectors were described by Equations (1)–(3), while the meridian patterns were described by a superposition of scattering from spherical domains and lamellar stacks (Equations (4)–(6)). The water domains were characterized by a spherical form factor combined with the hard-sphere structure factor, which is an approach that is usually employed for the interpretation of scattering data from spherical polymeric micelles [57,58], but is also applied for the characterization of ionic aggregates in PEMs [59,60]. Thus, four free parameters are used for describing the scattering from water domains in the Q range between 0.008 and 0.2 Å^−1^ according to Equations (1)–(3), namely the “forward scattering” (I_0_)^sph^ from the ensemble of the spherical water domains, the radius R of these domains, the hard sphere volume fraction η_HS_, and the hard-sphere radius R_HS_. Additionally, we added a Gaussian term for the description of the ionomer peak at high Q and the constant background (Equation (1)). The three parameters of the Gaussian function describing the ionomer peak—amplitude, width, and position—were left free during the fitting procedure, while the background was kept fixed, as given by the flat behavior of the scattering curves in the high Q range. The ionomer peak description was included in the model because of its presence in the meridian pattern, too, which will help for an accurate modeling of these data in a subsequent step. The 010 peak was excluded from the fitting procedure. Despite the multitude of parameters, we consider that the fitting procedure offers reliable results, because the two structures that are modeled appear at very different length scales, therefore without influencing one another to a significant extent. If only the form factor is used for modeling the water domains, the experimental data cannot be properly described. The weak correlation effects between the water domains seem to be a consequence of the high sulfonation degree of this sample, when water clusters are densely formed in the amorphous region. Detailed discussions of the formation, growth, and percolation of water clusters as a function of the hydration level and functionalization of PEMs can by found in [9,61].

The “forward scattering” from the ensemble of the spherical water domains and the size of these domains is of direct interest for the characterization of our system. We should note that a large polydispersity in size (σ_R_ ≈ 20%) of the water domains had to be considered in the fitting procedure in order to obtain a good fit in the Q region 0.1–0.2 Å^−1^. Knowing the size and the SLD of the scattering objects—the water domains, and the SLD of their environment—and the sulfonated segments of s-sPS (Table 1), we could extract information about the volume fraction ϕ_sph_ occupied by the scattering objects in the sample (Equation (1)), in a similar way to [62]. The volume fraction occupied by water in the whole amorphous region, (ϕ_water_)^amorphous^, is reported in Table 2. From the water volume fraction in the sample volume estimated from the interpretation of the (I_0_)^sph^, the reported value for each RH is obtained by taking into account the crystallinity of the film, which was estimated at 35%, and the fact that only the amorphous regions are hydrated.

The data measured on the meridian sector were modeled for a morphology consisting of oriented crystalline-amorphous lamellar stacks, which are “embedded” in a bulk amorphous environment (Figure 1). The scattering was described by combining Equations (1) and (4)–(6), and was superimposed over the scattering from water domains (including the ionomer peak contribution), which is isotropic and is known from the model interpretation of the equatorial data. Assuming a very large lateral extension of the lamellae, R_l_ > 1000 Å—thus out of the size domain that is covered by the SANS window—and a constant thickness of the crystalline lamellae d = 60 Å, which is an average value of what is reported in the literature for sPS crystals with different degrees of crystallinity and subjected to different treatments [63], only two free size parameters were used in the fitting procedure. These were, namely, the thickness of the interlamellar layer, L_b_, and the dispersion (smearing) parameter, σ_D_, of the interlamellar spacing, L_D_ = d + L_b_. As discussed in Section 2.3, the SLD of the crystalline and interlamellar amorphous layers, ρ_lam_ and ρ_inter-lam_, were considered free during the fitting procedure, while that of the bulk region ρ_bulk_ was considered that of the amorphous sPS (Table 1). Finally, the volume fraction of the lamellar stacks in Equation (1) was considered fixed and taken from the assumed crystalline degree of the material (35%).

All three experimental curves in Figure 6b were modeled simultaneously. Since the crystalline lamellae are not changing during hydration, the ρ_lam_ free parameter was considered the same for all curves, while the other free parameters were left to vary specifically to each RH condition. The model lines in Figure 6b describe rather well the experimental data, and the fitting procedure delivered the main parameters reported in Table 2. As we already noted, the sulfonation of the amorphous regions in the sPS film induced a swelling of the interlamellar domains in comparison with the non-sulfonated films, as reported in [28]. Therefore, the slightly larger value for the thickness of the interlamellar layer, L_b_, and resulting interlamellar spacing, L_D_, was obtained in our case. This quantitative analysis indicates a certain swelling of the interlamellar regions with increasing hydration, which was deduced from the slight increase in the thickness of the interlamellar layer L_b_. However, we should note that the model interpretation of the current data also indicates an increase in the smearing σ_D_ of the fitted interlamellar correlation distance between the oriented lamellae L_D_ = d + L_b_, which makes the actual swelling of the amorphous interlamellar layer difficult to assess.

To obtain semi-quantitative information about the volume fraction of water accumulated in the interlamellar amorphous region, the fitted SLD was further interpreted based on the assumptions made on the polymer and water volume fractions in these regions (Section 2.3). Thus, at a low hydration level, the water fraction in the interlamellar space is rather similar to that in the whole amorphous regions of the film sample. With increasing RH, the water domains grow in size and number, apparently (Table 2). From the evaluated values for (ϕ_water_)^amorphous^ and (ϕ_water_)^inter-lam^, we can conclude that the formation and growth of the water domains with increasing hydration seem to happen almost only in the amorphous bulk region, while the water volume fraction in the interlamellar amorphous layers remains quite constant. This may also explain the aspect of the ionomer peak (Figure 7), due to ionic clusters that remain dry still at high RH values. In addition, keeping in mind that the hydrated regions are characterized by a large polydispersity in size, we can assume that smaller water domains are present in the interlamellar regions compared to the bulk regions. These effects may be caused by the increased flexibility of the sPS chains in the bulk amorphous domains compared to that of the amorphous sPS chains between the crystalline lamellae, which favors the formation and growth of water domains mostly in the bulk amorphous region. For very high hydration levels, RH > 85%, a growth and percolation of water domains takes place in the bulk region, which ultimately leads to changes in the orientation and position of lamellar stacks, as reported before [25]. The preservation of the lamellar stacks arrangements even at a very high hydration level (saturation) is indicative of the lower water uptake in the interlamellar amorphous regions as in the bulk amorphous ones.

Finally, the fitting procedure delivered the value ρ_lam_ = 6.012 × 10^10^ cm^−2^ for the SLD of the crystalline lamellae, a value which is lower than that of crystalline sPS (Table 1). If we consider that the sPS lamellae are loaded with a protonated toluene guest, from the interpretation of the fitted value, we obtain a volume fraction of about 8.2% protonated toluene hosted between the sPS helices in the crystalline region, which is a value that is in very good agreement with what is reported in the literature [30].

### 3.4. SANS on Hydrated Films—Neutron Contrast Variation

**Figure 8:**
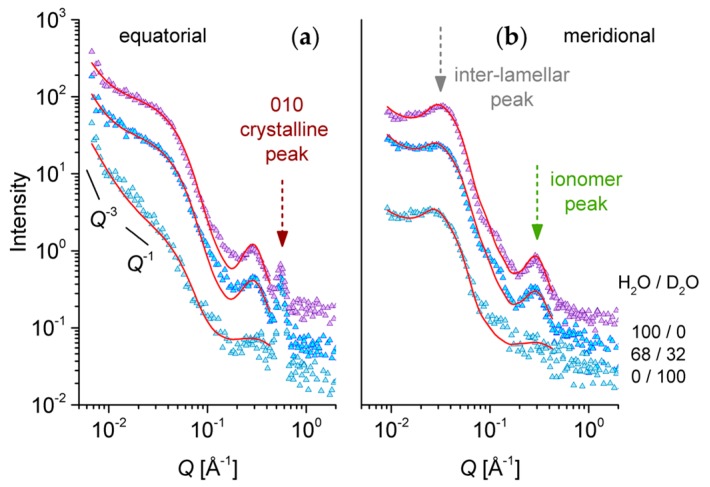
One-dimensional SANS patterns from the low sulfonated s-sPS film containing the co-crystalline δ-phase with protonated toluene and loaded with C60 fullerenes hydrated at relative humidity (RH) = 80% with different H^2^O/D^2^O ratios, as indicated in the right side of the plots. Experimental data (symbols) averaged over the equatorial (**a**) or meridian (**b**) directions are shown separately, with the lines corresponding to the model interpretation of the scattering profiles, as discussed in the text. The main structural features are indicated by arrows, while the solid lines in the panel (a) indicate the power law behavior of the scattering intensity in different Q ranges.

The equatorial and meridional scattering patterns from of the s-sPS film with a low degree of sulfonation (S = 19.3%) and lower crystallinity (22%) loaded with C60 fullerenes are presented in Figure 8. They were collected at a constant hydration level, RH = 80%, which was achieved using different H_2_O/D_2_O mixtures. Three neutron contrast conditions corresponding to the H_2_O/D_2_O ratios (vol%) of 100/0, 68/32, and 0/100 were investigated. The middle ratio corresponds to the matching SLD calculated for the sulfonic acid terminal group (SO_3_H). 

For the 100/0 H_2_O/D_2_O case, the same scattering features as in the case of the sample with a high degree of sulfonation (Figure 6) can be observed: the 010 crystalline peak that is revealed only in the equator direction, the isotropic ionomer peak that is visible in both the equatorial and meridian scattering patterns, and the interlamellar peak that is observed only in the meridian direction, at a lower Q value than the ionomer peak. The profile of the interlamellar correlation peak is not as strong as in the case of the sample with a higher sulfonation degree (Figure 6), which may be due to the lower crystallinity in the present sample. Unlike for the high sulfonation degree sample, in the present case, the scattering from the water domains (equatorial sectors) does not show a shoulder-like feature at around Q = 0.05 Å^−1^. Instead, a strong upturn appears toward the low Q region. A similar feature was observed in the very low Q region of the scattering patterns from highly sulfonated films [28]. The absence of the shoulder-like feature indicates that there is no correlation effect between the water domains. This may be due to the lower sulfonation degree in the present sample, which makes the water domains form and grow in the amorphous regions well separated from each other. On the other hand, the upturn at low Q, which appears stronger on the equator direction due to the stretching of a sample on the vertical axis, arises from the large-scale fractal-like character of the membranes. This feature is not visible toward low Q values in the patterns reported in Figure 6. This may be a consequence of the stronger correlation effects between the lamellae in the stack in that case: the strong structure factor peak induces an intensity drop toward low Q, and consequently, the intensity upturn is becoming observable at lower Q values. The intensity upturn at low Q values can be described by a power law [28,38], which should be added to the model equations that are used for fitting the experimental data, but is less important for the data interpretation in this work. The 70/30 H_2_O/D_2_O data show basically the same scattering features that are shown by the 100/0 H_2_O/D_2_O patterns, only the global intensity is lower, due to the lower contrast achieved between the hydrated and non-hydrated components of the film morphology. No matching of any scattering feature is visible. The data measured under the 0/100 H_2_O/D_2_O contrast show a different behavior from the other two contrast conditions. The first striking effect is the vanishing of the ionomer peak. We assume that the matching of the scattering properties of ionic clusters and surrounding water is achieved, which renders the correlation between the ionic clusters no longer visible. From this observation, a very important conclusion may be drawn: the ionic clusters that are promoting the water uptake by the membrane consist of an association of larger sections of neighboring s-sPS chains in the region of the benzene ring and the sulfonic acid terminal group, which are correlated over the distance 2π/Q_ion_. With increasing hydration, the water domain grows, and the correlation distance between these groups increases. The correlation effects are vanishing in the scattering patterns when the hydration medium has a similar SLD to that of the sulfonated sPS segment, which is roughly the D_2_O case. 

Another peculiarity of the scattering data in this contrast condition is the low Q behavior, where a Q^−1^ power law behavior of the scattered intensity may be roughly identified in the equatorial profile, rather than the spherical form factor profile combined with a low-Q steeper power law feature as for the other contrast conditions, or the case of the highly sulfonated sample [28]. The Q^−1^ power law behavior is indicative of the one-dimensional structures present in the sample. If we consider that the water accumulates along groups of elongated s-sPS chains in the amorphous region, this will highlight the hydrated regions as one-dimensional arrangements in contrast to the surrounding crystalline or non-sulfonated sPS environment, which is in agreement with the observed scattering behavior.

The interpretation of the experimental data was done in a similar way as for the sPS film with a higher sulfonation degree (Figure 6). The equatorial data were described by the combination of a spherical form factor of the water clusters (Equation (2)) and a Gaussian feature of the ionomer peak. An additional Q^−3^ power-law term was added to describe the low-Q data behavior. The meridian data were fitted by a superposition of the scattering features from the water clusters and the correlated lamellar stacks (Equations (4)–(6)). The modeling of the experimental data was quite successful, and has delivered the parameters reported in Table 2. For the description of the equatorial data, only a spherical form factor was considered, as no tracks of correlation between the water clusters were observed. The experimental data were separately fitted on the equatorial sectors first, to obtain the water domains parameters, and then on the meridian direction, simultaneously for all contrast conditions. The fitting procedure was carried out as discussed in Section 3.3. The L_b_ parameter was fitted, but kept the same for all contrast condition, since they were measured at the same RH. The low-Q power law behavior was considered only for the equatorial data interpretation, where it is more prominent due to the uniaxial sample deformation in the vertical direction. 

The volume fraction that is occupied by water in the whole sample is roughly the same for all contrast conditions, about 18%, which is to be expected since all measurements were carried out at the same RH.

From the fitted SLD of the interlamellar layers, semi-quantitative information about the volume fraction occupied by water in these regions could be obtained. Thus, the water occupies between 5% and 7%, as delivered by the fit of the 100/0 and 70/30 contrast conditions. The fitted results for the 0/100 contrast condition could not be further interpreted in a reliable way. A value with no physical meaning was obtained; therefore, we believe that in this case, a more specific model should be used for the interpretation of the experimental data. Nevertheless, all the values obtained for the lamellar stacks in the other two contrast conditions are consistent with each other. Therefore, the model interpretation of this data is considered realistic. Finally, the value ρ_lam_ = 6.003 × 10^10^ cm^−2^ for the SLD of the crystalline lamellae delivered a volume fraction of about 8.5% protonated toluene hosted between the sPS helices in the crystalline region, which again is a value that is in very good agreement with what is reported in the literature [30].

## 4. Conclusions

SANS with contrast variation was used to resolve the complex morphology of the sulfonated semi-crystalline syndiotactic polystyrene membranes at different hydration levels. Samples with different crystallinities were studied for different sulfonation degrees achieved by using the solid-state sulfonation procedure, which only affects the amorphous regions and leaves the crystallinity unchanged.

Fullerenes, which may improve the resistance to oxidation decomposition in PEMFC conditions, were incorporated in the membranes. Since no changes appear in the peak positions observed in the WAXD patterns, the fullerenes seem to be chiefly located in the amorphous regions of the samples. Apparently, the fullerenes do not influence the formation and evolution of the morphologies in the polymer films, as no significant differences were observed in the SANS patterns compared to the fullerenes free s-sPS membranes, which were investigated in a previous study. On the other hand, the scattering from fullerenes or fullerene assemblies dissolved in solution or an amorphous polymer environment is very weak [56], and provides a contribution to the measured scattering patterns that can be considered negligible compared to that from the dry or hydrated polymer morphologies in our samples. A separate study should be devoted to the behavior of fullerenes in these films, as long as the UV-Vis results also have not clearly revealed the absorption peaks specific to the fullerenes units, although the presence of fullerenes in the samples could be confirmed.

The formation of the crystalline regions in the membranes is a consequence of the initial exposure of the film samples to toluene in solution. Crystalline lamellae characterized by the co-crystalline δ-form of sPS with toluene molecules were formed. The exchange between deuterated and protonated toluene in the cavities between the sPS helices allowed for the variation of the neutron SLD of crystalline lamellae. The hydration of samples under controlled relative humidity affected only the sulfonated amorphous regions. The variation of the scattering properties of the hydrated domains was achieved using different H_2_O/D_2_O mixtures.

The use of uniaxially deformed films allowed the separation on different detection sectors of the scattering from structures of similar size but different orientation and functionality, which compose the dry and hydrated membrane morphologies. The SANS investigation carried out over a wide Q-range allowed the observation and characterization with models of structures that appear and evolve with increasing humidity at different length scales.

According to our qualitative and quantitative analysis, the hydration water is distributed to the same extent in the interlamellar and bulk amorphous regions at low hydrations, whereas with increasing the hydration level, the water accumulates predominantly in the bulk amorphous region. The contrast variation measurements revealed that the ionic clusters promoting the hydration and conductivity of the membranes consist not only of sulfonic groups, but also of segments of sPS that are affected by the sulfonation process. At very high hydration levels (saturation), the water domains evolve into water channels, which induce a displacement and change in orientation of lamellar stacks [28]. Nevertheless, the crystallinity, and hence the robustness of membrane, is preserved.

**Figure 9:**
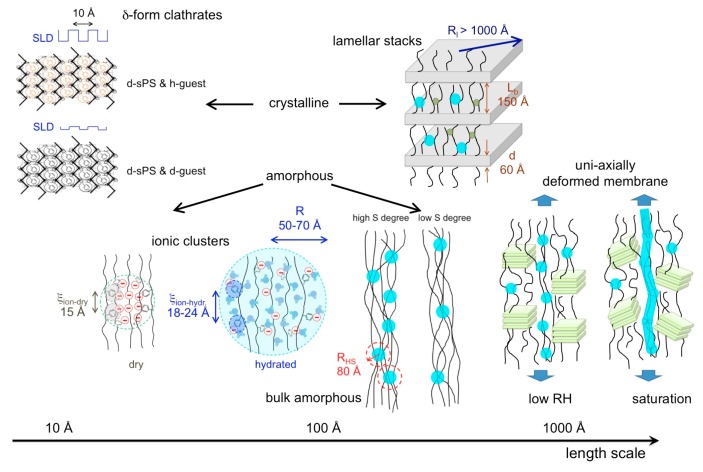
Structural levels that form on the length scale between nm and μm in dry and hydrated membranes based on semi-crystalline s-sPS, as identified and characterized by contrast variation SANS. The turquoise full circles distributed in the bulk and interlamellar amorphous regions represent the hydrated ionic clusters, while the green small dots in in the interlamellar amorphous layers are the still dry ionic clusters. At very high hydration levels (saturation), water channels are formed in the bulk amorphous regions [28].

A global picture of the multiple structural level character of the membrane morphology, as deduced from the SANS analysis, is reported in Figure 9. Knowing now in detail the morphology of the s-sPS polymer films in dry and hydrated states, the investigation of the influence of the temperature on the microstructure in such membranes under controlled humidity conditions will be the subject of a forthcoming study.

## Figures and Tables

**Table 1:** The calculated scattering length density (SLD) for different components of the sulfonated synditoactic polystyrene (s-sPS) films.

Compound	SLD, 10^10^ cm^−2^
sPS (crystalline)	6.47
sPS (amorphous)	6.00
s-sPS (amorphous)	6.34
–SO_3_H	1.32 (1.1 [36])
D_2_O	6.38
H_2_O	−0.56
d-Tol	5.66
h-Tol	0.94
C60	5.50
C70	5.67

**Table 2:** The structural and scattering parameters of the hydrated and lamellar morphologies delivered by the model interpretation of the experimental data according to Equations (1)–(7).

Sample	Condition	R, Å/R_HS_, Å	(ϕ_water_)^amorphous^, %	ρ_lam_, × 10^10^ cm^−2^	L_b_, Å/σ_D_, Å	ρ_inter-lam_, × 10^10^ cm^−2^	(ϕ_water_)^inter-lam^, %
S = 46.3%, with C70	RH = 50% 100 H_2_O/0 D_2_O	55.0/73.0	4.53	6.012	130.6/107.9	5.989	5.09
-	RH = 70% 100 H_2_O/0 D_2_O	58.8/77.0	7.45	6.012	151.6/114.7	5.983	5.17
-	RH = 80% 100 H_2_O/0 D_2_O	68.6/85.0	12.63	6.012	172.0/120.5	5.981	5.20
-	-	-	-	-	-	-	
S = 19.5%, with C60	RH = 80% 100 H_2_O/0 D_2_O	52/-	18.20	6.003	159.2/99.3	5.989	5.10
-	RH = 80 %68 H_2_O/32 D_2_O	52/-	17.85	6.003	155.6/106.7	5.993	7.20
-	-	-	-	-	-	-	-
-	RH = 80% 0 H_2_O/100 D_2_O	52/-	18.50	6.003	135.4/110.9	5.999	-
